# Evaluation of the accuracy of large language models in answering bone cancer-related questions

**DOI:** 10.3389/fpubh.2025.1719047

**Published:** 2025-12-05

**Authors:** Qilin Pan, Leiwen Huang, Ning Liu, Feixiang Lin, Shuxi Ye

**Affiliations:** Department of Spine Surgery, Ganzhou Hospital-Nanfang Hospital, Southern Medical, Ganzhou, Jiangxi, China

**Keywords:** large language models, Deepseek V3.1, ChatGPT 5, Grok 4, bone cancer, health information

## Abstract

**Introduction:**

Large Language Models (LLMs) excel at understanding medical terminology, parsing unstructured clinical data, and generating contextually relevant insights, emerging as transformative healthcare tools. Three leading LLMs—Deepseek, ChatGPT, and Grok—show great potential for medical education, clinical decision-making, and patient care. Bone cancer includes diverse primary and metastatic tumors, each with distinct diagnostic criteria, treatment pathways, and prognoses. Based on this guideline, this study assesses the accuracy of Deepseek V3.1, ChatGPT 5, and Grok 4 in addressing bone cancer-related questions.

**Methods:**

Based on the clinical guidelines for bone cancer released by the NCCN in April 2025, 52 questions related to bone cancer were developed. Researchers posed questions to Deepseek V3.1, ChatGPT 5, and Grok 4, and collected the data generated; each LLM was queried twice within a one-month period. The collected data were independently evaluated and scored by two bone cancer-treating specialists in accordance with the scoring criteria.

**Results:**

Among the answers to the 52 bone cancer-related questions, the probability of Deepseek V3.1, ChatGPT 5, and Grok 4 providing correct responses in both rounds was greater than 90%. Additionally, no correlation was observed between the LLMs’ scores, word count, and response times. The total scores of Deepseek V3.1, ChatGPT 5, and Grok 4 were 3.75 ± 0.71, 3.81 ± 0.6, and 0.87 ± 0.51, respectively. The word count of responses from Deepseek V3.1, ChatGPT 5, and Grok 4 was 546.56 ± 194.49, 367.02 ± 273.18, and 194.16 ± 197.07 words, respectively. The response times of Deepseek V3.1, ChatGPT 5, and Grok 4 were 11.83 ± 3.41 s, 1.52 ± 0.52 s and 42.48 ± 26.89 s, respectively. No statistically significant differences in scores were found for any of the LLMs between the two rounds. However, ChatGPT 5 showed a statistically significant difference in word count between the two rounds (360.12 ± 279.89 vs. 373.94 ± 268.86 words).

**Conclusion:**

When answering bone cancer-related questions, Deepseek V3.1, ChatGPT 5, and Grok 4 generally performed well. Specifically, when responding to questions about Ewing sarcoma, ChatGPT 5 and Grok 4 demonstrated higher accuracy than Deepseek V3.1. While each model has its own strengths and limitations, their collective potential to enhance medical knowledge and improve healthcare outcomes is undeniable.

## Introduction

Large Language Models (LLMs) are built on advanced deep learning and Natural Language Processing (NLP) technologies. They excel in understanding complex medical terminology, parsing unstructured clinical data, and generating contextually relevant insights, making them transformative tools in the healthcare field (1). Additionally, LLMs possess the ability to analyze massive datasets, including Electronic Health Records (EHRs), medical literature, and patient-generated text. For instance, LLMs have demonstrated notable proficiency in classifying injuries from emergency department records, which facilitates early risk detection and informs preventive measures (1–3). In clinical practice, LLMs are revolutionizing diagnostic and treatment decisions by interpreting complex medical data and generating actionable insights. For example, multimodal LLMs—such as the Medical Multimodal Large Language Model (Med-MLLM)—integrate visual and textual data to support tasks like disease reporting, diagnosis, and prognosis, even in scenarios with limited labeled data (e.g., rare diseases or emerging epidemics) (4).

Beyond clinical applications, LLMs are supporting medical training and knowledge dissemination by assisting with literature reviews, generating educational content, and answering complex medical questions—with models like Med-PaLM having demonstrated expert-level performance in medical licensing exams, which highlights their potential in this area (1, 5). Furthermore, LLMs are being explored for analyzing large datasets to identify patterns and relationships that might escape human researchers’ attention, particularly in neurology and other specialized fields (6). The potential of LLMs in healthcare is further underscored by their ability to generate human-like text, which enables applications such as patient communication, medical documentation, and even the creation of synthetic clinical cases for training purposes (7). However, their reliance on web-scraped data during training introduces a risk of propagating misinformation—especially in the healthcare field, where accuracy is critical (8). For instance, Med-PaLM 2 has demonstrated significant improvements over its predecessor, achieving high accuracy on medical question-answering datasets and gaining favor among physicians in pilot studies (9). Despite the promising outlook, the deployment of LLMs in traditional healthcare settings remains limited, and debates about the evidence required to validate their clinical use continue (10).

A key challenge in evaluating LLMs on bone cancer-related questions lies in the inherent complexity of the disease itself. Bone cancer encompasses a variety of primary and metastatic tumors, each with unique diagnostic criteria, treatment pathways, and prognostic implications. For instance, the differential diagnosis of bone lesions typically requires the integration of demographic, imaging, and histopathological features—a task that can be challenging even for experienced radiologists (11). However, LLMs, which rely on unstructured text data, must demonstrate performance comparable to or superior to traditional methods to be regarded as viable alternatives. Recent advances in LLMs have shown potential in addressing these challenges. For example, Med-PaLM 2—a state-of-the-art large language model tailored for medical applications—has achieved notable improvements in oncology-related medical question-answering tasks. It can generate accurate and clinically relevant responses to complex queries, a capability validated through extensive human evaluations. In some cases, physicians even prefer its answers over those provided by general practitioners (GPs) (9).

Integrating large language models (LLMs) such as DeepSeek, ChatGPT, and Grok into the domain of disease knowledge has demonstrated significant potential in enhancing medical education, clinical decision-making, and patient care. Powered by advanced Natural Language Processing (NLP) technology, these models are increasingly being used to answer complex medical questions, explain diagnostic scenarios, and provide insights into disease management. However, their performance varies across different medical fields, highlighting both their strengths and limitations in handling specialized knowledge (12–14). As a relatively new player in the LLM field, DeepSeek has shown potential in accurately answering disease-related queries. Its advanced reasoning capabilities, combined with a reinforcement learning framework, enable it to generate detailed responses to clinical questions. For instance, DeepSeek-R1 achieved a diagnostic accuracy rate of 91.6% when diagnosing oral diseases using text-based case descriptions—outperforming ChatGPT-4o (88.9%) and significantly surpassing the accuracy rate of human readers (47.8%) (15). ChatGPT, powered by OpenAI’s GPT-4 and GPT-5 models, has been extensively studied for its applications in disease knowledge and clinical scenarios. Similarly, in a study evaluating ChatGPT’s performance in the field of microbiology, its accuracy rate reached 71%, which was comparable to that of Google’s Gemini (70.5%); however, its performance varied across different subfields (e.g., general microbiology and applied microbiology) (16). Grok is another emerging LLM that has been evaluated alongside DeepSeek and ChatGPT in addressing high-complexity medical questions. In a study involving ophthalmological questions, Grok 3 achieved an accuracy rate of 69.2%, which was lower than that of DeepSeek-R1 (72.5%) and OpenAI’s o1 Pro (83.4%) (17).

However, for Deepseek, ChatGPT, and Grok—three of the world’s most popular Large Language Models (LLMs)—there is currently a lack of relevant research exploring their accuracy in answering bone cancer-related questions. The guidelines released by the National Comprehensive Cancer Network (NCCN) in April 2025 focus primarily on Ewing sarcoma and osteosarcoma (18). Using this guideline as the standard, this study investigates the accuracy of Deepseek V3.1, ChatGPT 5, and Grok 4 in answering questions related to bone cancer.

## Methods

**Figure 1:**
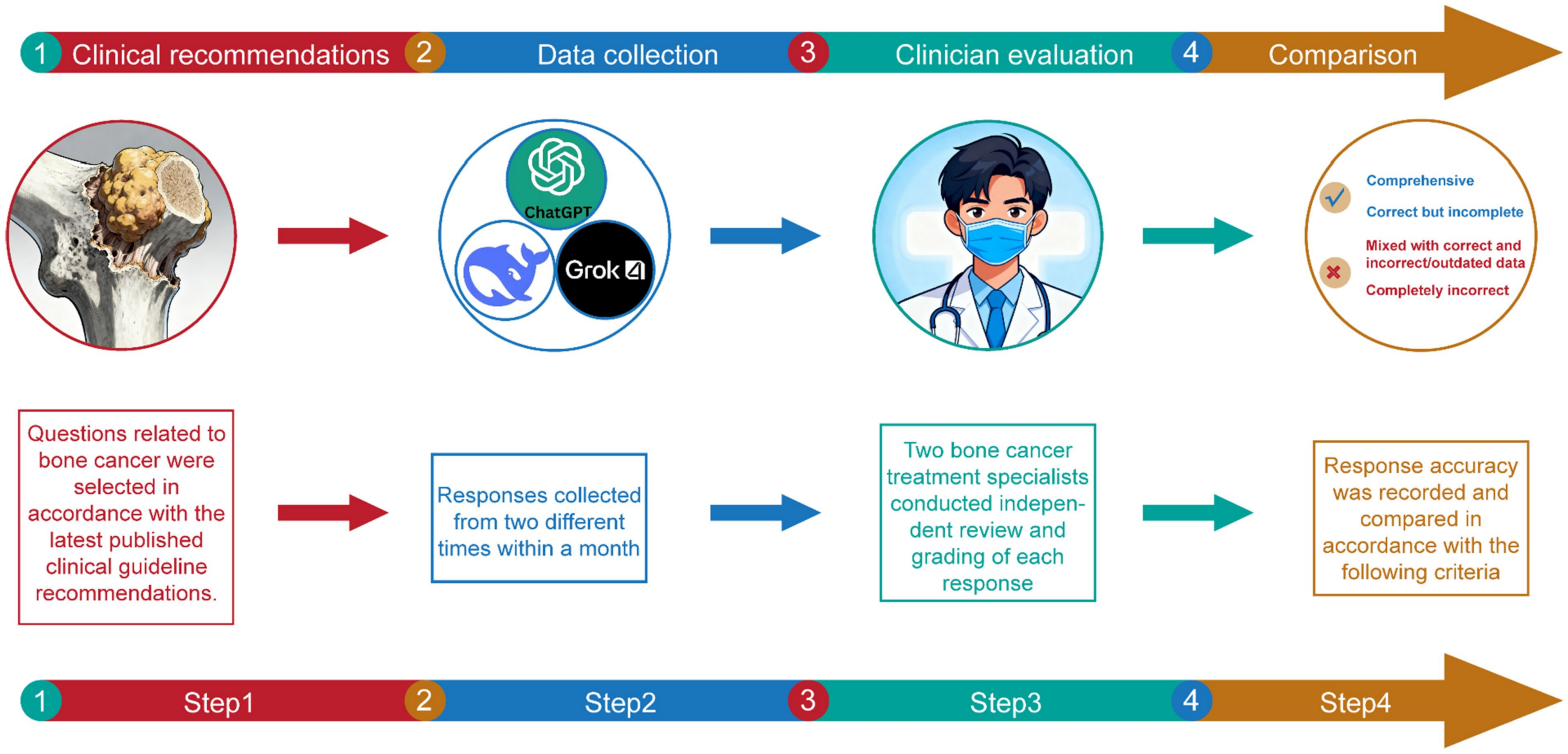
The flow chart of this study.

The research workflow of this study is shown in Figure 1, and it consists of four main steps: Clinical recommendations, data collection, clinician evaluation, and comparison.

### Clinical recommendations and data collection

The National Comprehensive Cancer Network (NCCN) released the clinical guideline on bone cancer—Bone Cancer, Version 2.2025, NCCN Clinical Practice Guidelines in Oncology—in April 2025 (18). This guideline is primarily divided into three sections: Overview, Ewing Sarcoma, and Osteosarcoma. Based on this guideline, we developed 52 questions related to bone cancer, with 15 questions derived from the Overview section, 19 from the Ewing Sarcoma section, and 18 from the Osteosarcoma section. These questions cover various aspects of bone cancer, including its etiology, pathology, diagnosis, and treatment. Questions were selected following the presentation order of the NCCN Clinical Practice Guidelines: First, in the Overview section, questions were designed based on consensus-based content, such as “In adults, what is the most common primary bone cancer?” Second, Sections 2 and 3 (Ewing sarcoma and Osteosarcoma) cover disease-specific basic knowledge, with questions including “What are the characteristic gene fusions in Ewing sarcoma?” and “What are the common predilection sites of high-grade intramedullary osteosarcoma?” Additionally, questions were formulated targeting strongly recommended recommendations by experts, such as the guideline statement: “Wide excision is recommended as the primary treatment for patients with low-grade (intramedullary and surface) osteosarcomas and periosteal lesions.” Data collection was conducted in September 2025. The 52 questions were used to query the LLMs twice independently, and relevant data were collected during this process. Detailed data results are available in the Supplementary materials. Manual measurement was employed in this study: the start time was defined as the moment the query was input into the LLM, and the end time as the moment the model generated a complete response. All LLM response time measurements were conducted under a unified hardware and network environment.

### Clinician evaluation and comparison

The responses generated by the LLMs were independently evaluated and scored by two clinical specialists who treat bone cancer. The scoring criteria from a previous study were adopted (19): (1) Comprehensive (4 points); (2) Correct but incomplete (3 points); (3) Mixed with correct and incorrect/outdated data (2 points); and (4) Completely incorrect (1 point). A strict double-blinded design was adopted in this study: clinical evaluators were neither aware of the model that generated each response (with model identifiers such as name and version concealed) nor the corresponding test rounds (e.g., initial run/repeated runs). All LLM outputs were standardized with random codes (e.g., “1 ~ 52”) replacing model information and round labels, allowing evaluators to only access the question stems and response content without any potential bias from external information. For the evaluation process: Two attending physicians with more than 5 years of clinical experience and proficiency in NCCN guidelines were selected, and neither had participated in the prior question bank design nor model testing of this study. During the formal scoring phase, the two evaluators independently completed the scoring of all responses without communication. The scoring results were directly entered into a dedicated data sheet to avoid mutual interference. The Cohen’s Kappa coefficient for each question is available in Supplementary material S1. Additionally, Cohen’s kappa was used to assess the level of inter-rater agreement between the two scorers, with a result of 0.807.

### Statistical analysis

Statistical analyses and graphical visualization were performed using R (version 4.5.0), Origin 2024, and GraphPad Prism (version 9.3.0). *T*-test or Mann–Whitney U test was used to analyze the comparison between the two groups. One-way analysis of variance (ANOVA) (with Mann–Whitney U test or mixed methods) was used to compare multiple groups. *p*-values less than 0.05 were considered statistically significant. The weighted Cohen’s Kappa test was employed for pairwise per-question scoring by two evaluators, with quadratic weights selected to reflect the hierarchical differences among ordinal categories. Spearman’s rank correlation analysis was used for correlation assessment. Per-question LLM-guideline alignment score: A quantitative measure of consistency between a single evaluator’s rating and the guideline for an individual LLM response, serving as the foundational data for subsequent comprehensive analyses.

Per-question interrater reliability (weighted Cohen’s Kappa): Indicator definition: Quantifies the consistency of scores between any two evaluators for each individual question. Statistic for inter-model difference testing (Kruskal–Wallis H): A nonparametric test statistic that quantifies the distribution differences in comprehensive scores across multiple groups (three LLMs).

## Results

### Basic scoring results of LLMs’ responses to bone cancer-related questions and correlation assessment

**Figure 2:**
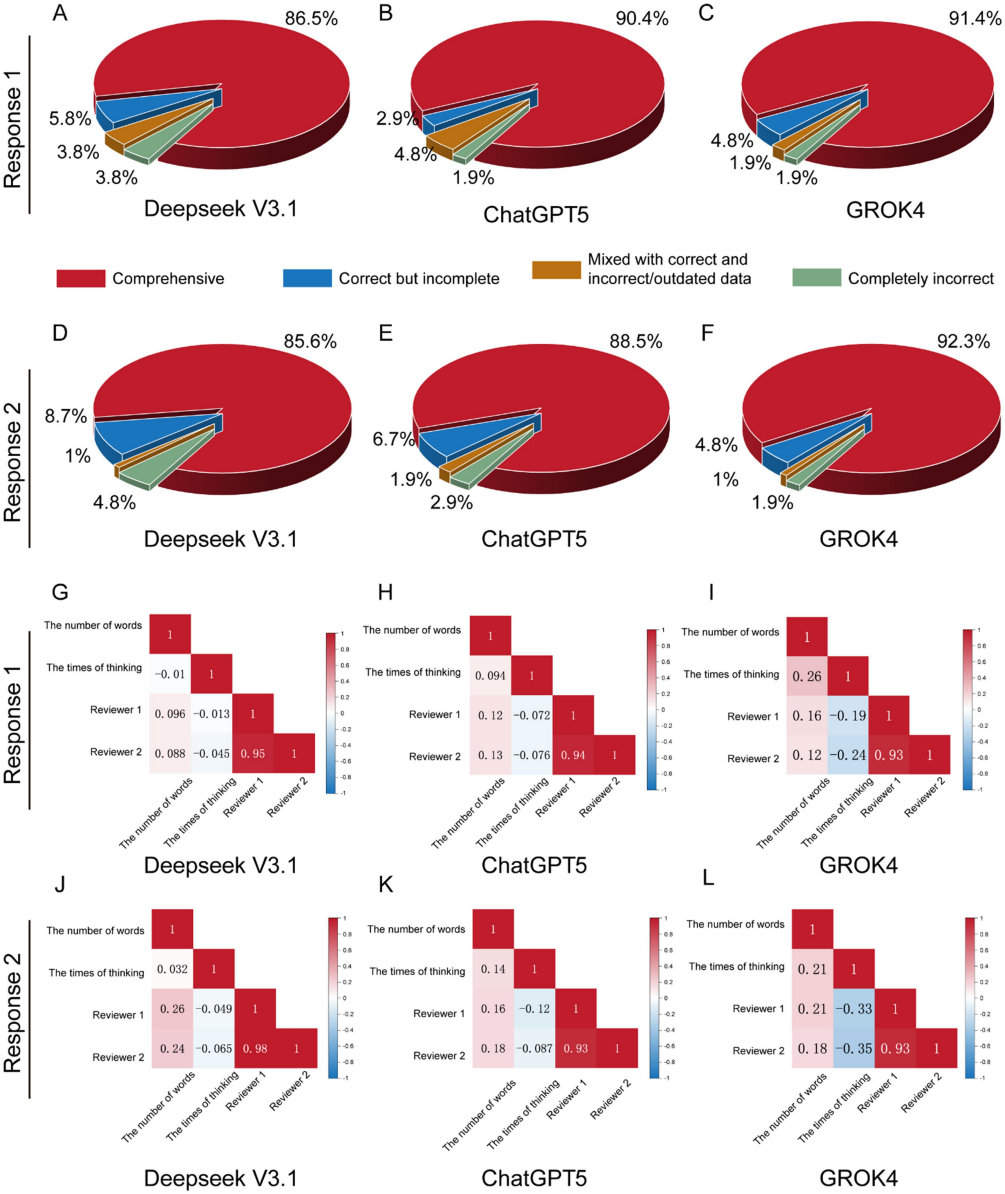
Basic scoring results of LLMs’ responses to bone cancer-related questions and correlation assessment. In the first round of runs, the score distributions of Deepseek V3.1 **(A)**, ChatGPT 5 **(B)**, and Grok 4 **(C)** are presented. In the second round of runs, the score distributions of Deepseek V3.1 **(D)**, ChatGPT 5 **(E)**, and Grok 4 **(F)** are shown. For the first round of runs, an analysis of the correlations between the number of words, response times, and scores is conducted for Deepseek V3.1 **(G)**, ChatGPT 5 **(H)**, and Grok 4 **(I)**. For the second round of runs, an analysis of the correlations between the number of words, response times, and scores is performed for Deepseek V3.1 **(J)**, ChatGPT 5 **(K)**, and Grok 4 **(L)**.

Figure 2A shows the scoring results of Deepseek V3.1 in the first round of questioning. “Comprehensive” accounts for 86.5%, “Correct but incomplete” accounts for 5.8%, “Mixed with correct and incorrect/outdated data” accounts for 3.8%, and “Completely incorrect” accounts for 3.8%. Figure 2B shows the scoring results of ChatGPT 5 in the first round of questioning. “Comprehensive” accounts for 90.4%, “Correct but incomplete” accounts for 2.9%, “Mixed with correct and incorrect/outdated data” accounts for 4.8%, and “Completely incorrect” accounts for 1.9%. Figure 2C shows the scoring results of Grok 4 in the first round of questioning. “Comprehensive” accounts for 91.4%, “Correct but incomplete” accounts for 4.8%, “Mixed with correct and incorrect/outdated data” accounts for 1.9%, and “Completely incorrect” accounts for 1.9%. The probabilities of Deepseek V3.1, ChatGPT 5, and Grok 4 providing correct responses (combining “Comprehensive” and “Correct but incomplete”) are 92.4%, 93.3%, and 96.2%, respectively.

In the second round of questioning, Figure 2D presents the scoring results of Deepseek V3.1: Comprehensive: 85.6%, Correct but incomplete: 8.7%, Mixed with correct and incorrect/outdated data: 1%, and completely incorrect: 4.8%. Figure 2E shows the scoring results of ChatGPT 5: Comprehensive: 88.5%, Correct but incomplete: 6.7%, Mixed with correct and incorrect/outdated data: 1.9%, and completely incorrect: 2.9%. Figure 2F displays the scoring results of Grok 4: Comprehensive: 92.3%, Correct but incomplete: 4.8%, Mixed with correct and incorrect/outdated data: 1%, and completely incorrect: 1.9%. The probabilities of Deepseek V3.1, ChatGPT 5, and Grok 4 providing correct responses (combining “Comprehensive” and “Correct but incomplete”) are 94.3%, 95.2%, and 97.1%, respectively.

We also collected the number of words and response times of the LLMs’ answers during data collection, and explored whether there was a correlation between these metrics and the scores. Figures 2G,J show the correlations between Reviewer 1, Reviewer 2, the number of words, and response time in Deepseek V3.1’s answers in the first and second rounds, respectively. The correlation coefficients between Reviewer 1 and the number of words were 0.096 and 0.26, respectively; and those between Reviewer 1 and response time were −0.013 and −0.049, respectively. The correlation coefficients between Reviewer 2 and the number of words were 0.088 and 0.24, respectively; and those between Reviewer 2 and response time were −0.045 and −0.065, respectively. The correlation coefficients between the number of words and response time were −0.01 and 0.032, respectively. Figures 2H,K show the correlations between Reviewer 1, Reviewer 2, the number of words, and response time in ChatGPT 5’s answers in the first and second rounds, respectively. The correlation coefficients between Reviewer 1 and the number of words were 0.12 and 0.16, respectively; and those between Reviewer 1 and response time were −0.072 and −0.12, respectively. The correlation coefficients between Reviewer 2 and the number of words were 0.13 and 0.18, respectively; and those between Reviewer 2 and response time were −0.076 and −0.087, respectively. The correlation coefficients between the number of words and response time were 0.094 and 0.14, respectively. Figures 2I,L show the correlations between Reviewer 1, Reviewer 2, the number of words, and response time in Grok 4’s answers in the first and second rounds, respectively. The correlation coefficients between Reviewer 1 and the number of words were 0.16 and 0.21, respectively; and those between Reviewer 1 and response time were −0.19 and −0.33, respectively. The correlation coefficients between Reviewer 2 and the number of words were 0.12 and 0.18, respectively; and those between Reviewer 2 and response time were −0.24 and −0.35, respectively. The correlation coefficients between the number of words and response time were 0.26 and 0.21, respectively.

### Comparison of scores, number of words, and response times among LLMs

**Figure 3:**
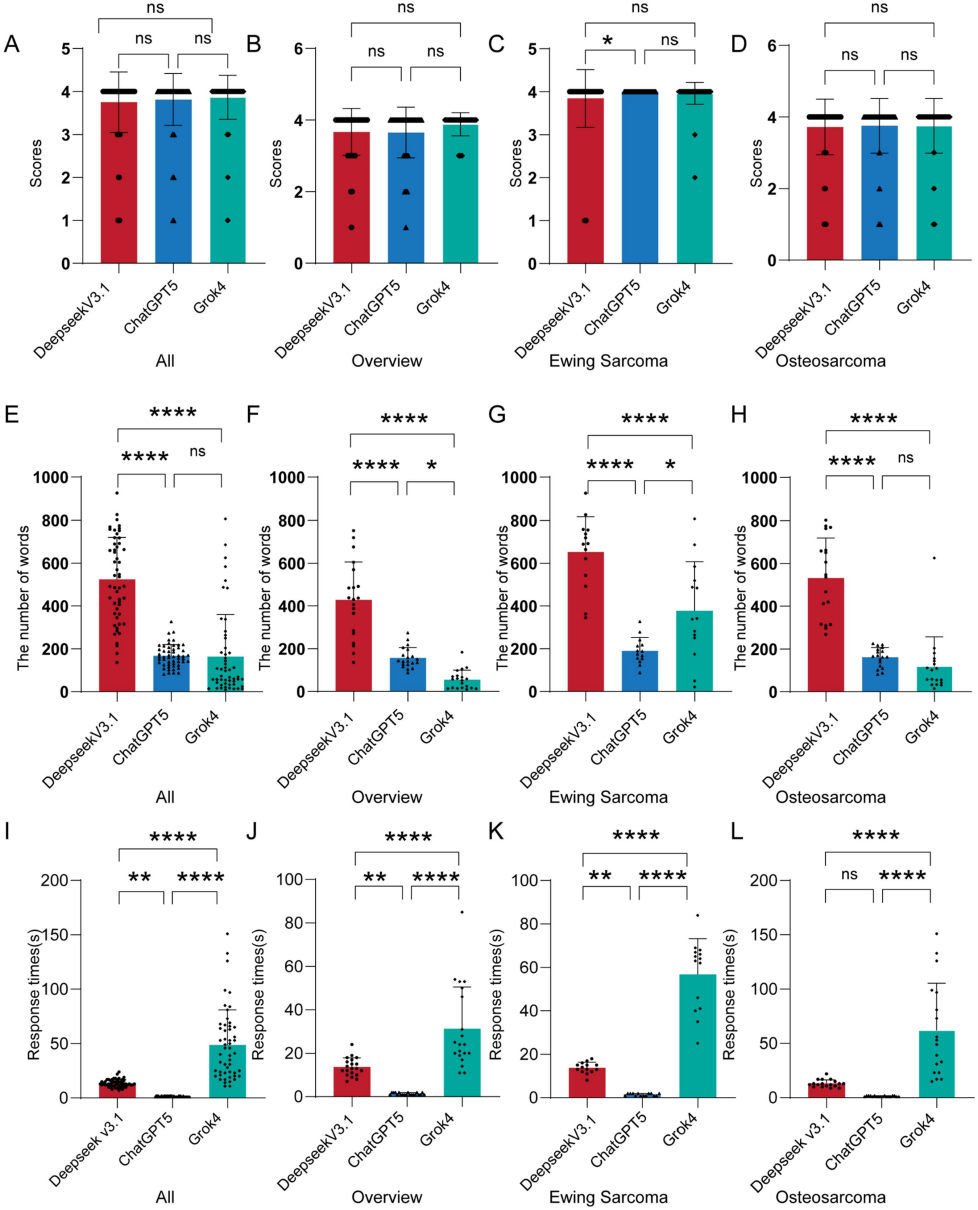
Comparison of scores, number of words, and response times among LLMs. Comparisons of scores among Deepseek V3.1, ChatGPT 5, and Grok 4 are conducted in four aspects: total scores **(A)**, Overview **(B)**, Ewing sarcoma **(C)**, and Osteosarcoma **(D)**. Comparisons of the number of words in responses among Deepseek V3.1, ChatGPT 5, and Grok 4 are performed in four aspects: total number of words **(E)**, Overview **(F)**, Ewing sarcoma **(G)**, and Osteosarcoma **(H)**. Comparisons of response times among Deepseek V3.1, ChatGPT 5, and Grok 4 are carried out in four aspects: total response times **(I)**, Overview **(J)**, Ewing sarcoma **(K)**, and Osteosarcoma **(L)**. ns, nonsignificant; *p < 0.05; **p < 0.01; ***p < 0.001; ****p < 0.0001.

We compared the total scores, the number of words, and response times of Deepseek V3.1, ChatGPT 5, and Grok 4. Regarding total scores, as shown in Table 1 and Figure 3A, the total scores of Deepseek V3.1, ChatGPT 5, and Grok 4 were 3.75 ± 0.71, 3.81 ± 0.6, and 0.87 ± 0.51, respectively, with no significant differences observed. Additionally, when answering questions in the Overview section (see Table 1; Figure 3B), the total scores of Deepseek V3.1, ChatGPT 5, and Grok 4 were 3.67 ± 0.66, 33.65 ± 0.71, and 3.89 ± 0.32, respectively, and no significant differences were found. Furthermore, in terms of answering questions about Ewing Sarcoma (see Table 1; Figure 3C), the total scores of Deepseek V3.1, ChatGPT 5, and Grok 4 were 3.84 ± 0.67, 4 ± 0, and 3.96 ± 0.26, respectively—with ChatGPT 5 achieving a significantly higher score than Deepseek V3.1. Finally, when answering questions about osteosarcoma (see Table 1; Figure 3D), the total scores of Deepseek V3.1, ChatGPT 5, and Grok 4 were 3.72 ± 0.77, 3.75 ± 0.76, and 3.75 ± 0.76, respectively.

**Table 1:** Comparison of scores, number of words, and response times among Deepseek V3.1, ChatGPT 5 and Grok 4.

Assessment dimensions	Domain	Deepseek V3.1	ChatGPT 5	Grok 4
Scores Mean (±sd)	ALL	3.75 ± 0.71	3.81 ± 0.6	3.87 ± 0.51
	Overview	3.67 ± 0.66	3.65 ± 0.71	3.89 ± 0.32
	Ewing sarcoma	3.84 ± 0.67	4 ± 0	3.96 ± 0.26
	Osteosarcoma	3.72 ± 0.77	3.75 ± 0.76	3.75 ± 0.76
The number of words Mean (±sd)	ALL	546.56 ± 194.49	367.02 ± 273.18	194.16 ± 197.07
	Overview	446.53 ± 168.09	219.83 ± 158.27	59.03 ± 40.12
	Ewing sarcoma	627.68 ± 171.61	446.47 ± 271.97	317.03 ± 217.32
	Osteosarcoma	531.94 ± 181.73	160.94 ± 44.60	119.11 ± 134.21
Response time Mean (±sd)	ALL	11.83 ± 3.41	1.52 ± 0.52	42.48 ± 26.89
	Overview	12.3 ± 4.28	1.53 ± 0.51	33.4 ± 18.80
	Ewing sarcoma	11.24 ± 2.80	1.55 ± 0.55	46.39 ± 17.62
	Osteosarcoma	12.06 ± 3.17	1.47 ± 0.51	45.92 ± 37.50

After counting the number of words in the LLMs’ answers, overall (Figure 3E), the number of words in the answers of Deepseek V3.1, ChatGPT 5, and Grok 4 was 546.56 ± 194.49, 367.02 ± 273.18, and 194.16 ± 197.07, respectively. For questions related to the Overview section (Figure 3F), the number of words in the answers of Deepseek V3.1, ChatGPT 5, and Grok 4 was 446.53 ± 168.09, 219.83 ± 158.27, and 59.03 ± 40.12, respectively. For questions related to Ewing Sarcoma (Figure 3G), the number of words in the answers of Deepseek V3.1, ChatGPT 5, and Grok 4 was 627.68 ± 171.61, 446.47 ± 271.97, and 317.03 ± 217.32, respectively. For questions related to Osteosarcoma (Figure 3H), the number of words in the answers of Deepseek V3.1, ChatGPT 5, and Grok 4 was 531.94 ± 181.73, 160.94 ± 44.60, and 119.11 ± 134.21, respectively. We found that Deepseek V3.1 had the highest number of words in its answers.

Additionally, we also recorded the response times of the LLMs when answering questions (unit: seconds). Overall (Figure 3I), the response times of Deepseek V3.1, ChatGPT 5, and Grok 4 were 11.83 ± 3.41, 1.52 ± 0.52, and 42.48 ± 26.89, respectively. For questions related to the Overview section (Figure 3J), the response times of Deepseek V3.1, ChatGPT 5, and Grok 4 were 12.3 ± 4.28, 1.53 ± 0.51, and 33.4 ± 18.80, respectively. For questions related to Ewing Sarcoma (Figure 3K), the response times of Deepseek V3.1, ChatGPT 5, and Grok 4 were 11.24 ± 2.80, 1.55 ± 0.55, and 46.39 ± 17.62, respectively. For questions related to Osteosarcoma (Figure 3L), the response times of Deepseek V3.1, ChatGPT 5, and Grok 4 were 12.06 ± 3.17, 1.47 ± 0.51, and 45.92 ± 37.50, respectively.

### Comparison of scores, number of words, and response times response 1 and response 2

**Figure 4:**
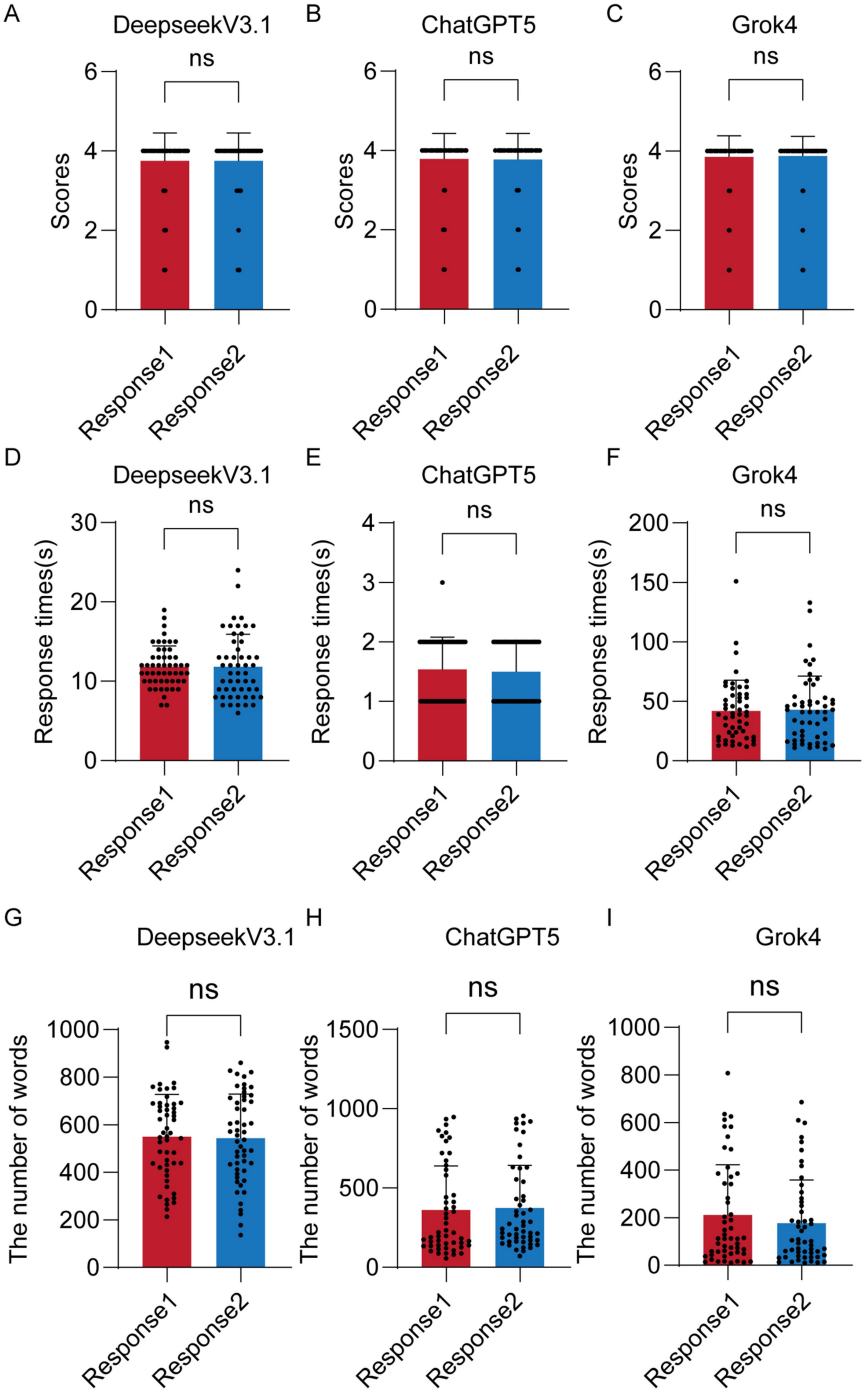
Comparison of scores, number of words, and response times between response 1 and response 2. Comparison of scores between the two runs for Deepseek V3.1 **(A)**, ChatGPT 5 **(B)**, and Grok 4 **(C)**. Comparison of response times between the two runs for Deepseek V3.1 **(D)**, ChatGPT 5 **(E)**, and Grok 4 **(F)**. Comparison of the number of words between the two runs for Deepseek V3.1 **(G)**, ChatGPT 5 **(H)**, and Grok 4 **(I)**. ns, nonsignificant; *p < 0.05.

Table 2 presents the comparison of scores, number of words, and response times of LLMs across different responses. In the first round of questioning: The scores of Deepseek V3.1, ChatGPT 5, and Grok 4 were 3.75 ± 0.71, 3.82 ± 0.60, and 3.86 ± 0.53, respectively; The number of words in their answers was 549.79 ± 183.42, 360.12 ± 279.89, and 211.23 ± 211.84, respectively; Their response times were 11.83 ± 2.61, 1.54 ± 0.54, and 41.96 ± 25.88, respectively. In the second round of questioning: The scores of Deepseek V3.1, ChatGPT 5, and Grok 4 were 3.75 ± 0.71, 3.80 ± 0.61, and 3.88 ± 0.50, respectively; The number of words in their answers was 543.33 ± 185.93, 373.94 ± 268.86, and 177.10 ± 181.57, respectively; Their response times were 11.83 ± 4.08, 1.50 ± 0.50, and 43 ± 28.10, respectively. As shown in Figures 4A–C, when comparing scores between Response 1 and Response 2, Deepseek V3.1, ChatGPT 5, and Grok 4 all showed no statistically significant differences. As indicated in Figures 4D–F, in terms of response times, only Deepseek V3.1 exhibited a significant difference between the two rounds of questioning. As seen in Figures 4G–I, regarding the number of words across all responses, only ChatGPT 5 showed a significant difference between the two rounds.

**Table 2:** Comparison of scores, number of words, and response times between response 1 and response 2.

Assessment dimensions	LLMs	Response 1	Response 2
Scores Mean (±sd)	Deepseek V3.1	3.75 ± 0.71	3.75 ± 0.71
	ChatGPT 5	3.82 ± 0.60	3.80 ± 0.61
	Grok 4	3.86 ± 0.53	3.88 ± 0.50
The number of words Mean (±sd)	Deepseek V3.1	549.79 ± 183.42	543.33 ± 185.93
	ChatGPT 5	360.12 ± 279.89	373.94 ± 268.86
	Grok 4	211.23 ± 211.84	177.10 ± 181.57
Response time Mean (±sd)	Deepseek V3.1	11.83 ± 2.61	11.83 ± 4.08
	ChatGPT 5	1.54 ± 0.54	1.50 ± 0.50
	Grok 4	41.96 ± 25.88	43 ± 28.10

## Discussion

LLMs can also enhance patient education and facilitate shared decision-making by providing accessible, evidence-based information on osteosarcoma and Ewing sarcoma. For instance, LLMs can generate personalized educational materials that explain treatment options, potential side effects, and long-term outcomes in plain language. This enables patients and their families to make informed decisions about their care, thereby improving treatment adherence and overall satisfaction (20, 21). In this study, we investigated the accuracy of Deepseek V3.1, ChatGPT 5, and Grok 4 in answering bone cancer-related questions. The study showed that these three models achieved high accuracy in the two rounds of answering bone cancer-related questions, with the probability of providing correct responses (combining “Comprehensive” and “Correct but incomplete”) all exceeding 90%. Subsequently, we conducted a correlation analysis of scores, the number of words, and response times, and found low correlation coefficients among them—indicating no association between accuracy, the number of words, and response times. Next, we compared Deepseek V3.1, ChatGPT 5, and Grok 4 across three aspects: scores, the number of words, and response times. In terms of scores, when answering Ewing sarcoma-related questions, ChatGPT 5 achieved a significantly higher score than Deepseek V3.1; however, there were no statistically significant differences among the three models when answering other types of questions. In terms of the number of words, Deepseek V3.1 had a significantly higher number of words in its answers than ChatGPT 5 and Grok 4. In terms of response times, Grok 4 took the longest time to answer questions, while ChatGPT 5 had the shortest response time.

The assessment of Large Language Models (LLMs) in terms of their accuracy in addressing disease-related knowledge has become a key research area, especially given their growing integration into healthcare and medical education. Recent studies have shown that LLMs such as ChatGPT-4, BARD, and BingAI demonstrate varying levels of proficiency when responding to clinical queries, with their performance often depending on the complexity and specificity of the questions posed (22–24). For example, in the field of ophthalmology, LLMs achieved an accuracy rate of up to 82.9% in postgraduate professional exams—without the need for prompt or instruction fine-tuning—and outperformed traditional evaluation benchmarks (23).

This highlights their potential as tools for supporting medical education and clinical decision-making. However, their performance is not entirely reliable, as evidenced by inconsistent responses to queries about complex or rare diseases—a problem that may pose a risk of misinformation (24). In this study, we found that there were certain discrepancies between the responses of LLMs and expert consensus for some questions. For Question 15 (For Ewing Sarcoma patients who have undergone wide resection with negative margins, can adjuvant chemotherapy alone be used?), the responses of ChatGPT 5 and Grok 4 aligned with expert consensus, while Deepseek V3.1’s response differed from the clinical guidelines. The guidelines state that adjuvant chemotherapy alone is recommended for patients who have undergone wide excision with negative margins. In contrast, Deepseek V3.1’s response was: “No, adjuvant chemotherapy alone is not considered sufficient.” For Question 21 (What is the most common predilection site of periosteal osteosarcoma?), the responses of Deepseek V3.1, ChatGPT 5, and Grok 4 all diverged from the clinical guidelines. The clinical guidelines indicate: “Periosteal osteosarcomas are intermediate-grade lesions, showing partial cartilaginous differentiation, and most often involve the femur, followed by the tibia.” However, the responses of Deepseek V3.1, ChatGPT 5, and Grok 4 all stated: “The most common predilection site of periosteal osteosarcoma is the tibia.” LLMs can serve as valuable resources for disseminating medical information. Nevertheless, in scenarios requiring high medical accuracy—such as diagnosing complex conditions or interpreting radiological reports—their limitations become prominent, and their performance often fails to match the level of human experts (25). A key challenge in assessing the accuracy of LLMs lies in the variability of their responses to complex and nuanced medical questions. For instance, while LLMs perform well when answering straightforward, fact-based questions, their performance declines when faced with open-ended or contextually complex questions—such as those involving differential diagnosis or rare diseases (26). The accuracy of LLMs in disease-related knowledge is also influenced by the quality and timeliness of their training data. Many LLMs, including ChatGPT-3.5 and GPT-4, are trained on data up to 2021, which limits their ability to provide up-to-date information on emerging diseases or the latest medical advancements (27, 28). Models with continuous updates, such as Bard, offer more timely information, but may face reproducibility issues due to their constantly changing nature (26). This temporal limitation poses significant risks in clinical settings: outdated or incomplete information can lead to the danger of misleading information, as observed in 2.8% to 18.9% of LLM-generated responses in clinical case studies on urology topics (24). Another critical aspect of accuracy assessment is the potential bias in the responses generated by LLMs. Studies have shown that LLMs may produce medically unjustified recommendations influenced by patients’ sociodemographic characteristics, such as race, income level, or sexual orientation (29).

Despite the fact that Deepseek V3.1, ChatGPT 5, and Grok 4 performed well in answering bone cancer-related questions in this study, they still produced incorrect answers. This indicates that limitations remain in the application of LLMs in the medical field—for example, the timely update of data and information, and the reliability of online text information sources. Additionally, this study has several limitations: (1) The use of a single language and only two independent runs make it difficult to fully characterize the stochastic variability and prompt sensitivity of modern LLMs. Thus, future studies should further conduct multilingual repeated tests, with a core focus on “verifying the matching degree between clinical questions and guidelines.” (2) The core focus of this study is “evaluating the basic performance and potential of general-purpose LLMs in clinical guideline-matching tasks,” focusing on mainstream general-purpose models without specialized medical fine-tuning, and medically fine-tuned LLMs were not included.

Therefore, we believe that future research should focus on developing open-access LLMs specifically trained on real medical data to improve their reliability, and explore mechanisms to estimate and communicate uncertainty in their responses (26). Additionally, the attribution of responsibility for errors or inaccuracies in responses generated by LLMs remains unclear, which necessitates the development of ethical frameworks and regulatory guidelines to regulate their use in healthcare.

## Conclusion

Overall, this study demonstrates that when evaluated against the recently published clinical guidelines for bone cancer, Deepseek V3.1, ChatGPT 5, and Grok 4 all demonstrated overall good performance in answering bone cancer-related questions. Specifically, when answering Ewing sarcoma-related questions, ChatGPT 5 and Grok 4 achieved higher accuracy than Deepseek V3.1. While each model has its own strengths and limitations, their collective potential to advance medical knowledge and improve healthcare outcomes is undeniable. As these models continue to evolve, they hold the promise of revolutionizing the way medical knowledge is accessed, interpreted, and applied in clinical practice.

## Data Availability

The original contributions presented in the study are included in the article/Supplementary material, further inquiries can be directed to the corresponding author.
